# Exceptional X-Ray contrast: Radiography imaging of a Middle Triassic mixosaurid from Svalbard

**DOI:** 10.1371/journal.pone.0285939

**Published:** 2023-05-31

**Authors:** Victoria S. Engelschiøn, Aubrey J. Roberts, Ruben With, Øyvind Hammer

**Affiliations:** 1 Natural History Museum, University of Oslo, Oslo, Norway; 2 Department of Earth Science, Natural History Museum, London, United Kingdom; 3 Museum of Cultural History, University of Oslo, Oslo, Norway; Chinese Academy of Sciences, CHINA

## Abstract

The black shales of the Middle Triassic Botneheia Formation in Svalbard are known for their fossil richness with abundant ichthyosaur remains and beds of the bivalve *Daonella*. Vertebrate remains from the Muen Mountain on Edgeøya are shown to have exceptional X-ray contrast due to a combination of sulphide and sulphate permineralisation and pseudomorphing. Radiography imaging of a previously described specimen, PMO 219.250, revealed new and spectacular details such as more carpals, teeth, and skull sutures. Teeth and skull characters are taxonomically significant. supporting the referral of PMO 219.250 to *Phalarodon* and further suggesting an affinity to *P*. *atavus*. Three sulphur phases were identified, with the sulphide sphalerite (ZnS) being the highest temperature phase, followed by the sulphate baryte (BaSO_4_), and the sulphide pyrite (FeS_2_). Sulphate permineralisation is also seen in specimens from the Upper Jurassic on Svalbard. We suggest that sulphur-rich fluids have flowed and dissolved barium from the shales and deposited the sphalerite and baryte, and that this could be linked to the Cretaceous HALIP. The Jurassic specimens are only permineralised by baryte, while the Triassic specimens have also been permineralised, but mainly pseudomorphed by baryte with crystals of sphalerite. Lithology differences appear to have controlled the compaction of the Triassic specimens, while the Jurassic specimens have retained their three-dimensional shape due to the baryte emplacement relatively earlier in their depositional history. Although soft tissues are not preserved, the excellent X-ray contrast in the Middle Triassic specimens is reminiscent of pyritised fossil sites such as the Hunsrück Slate (Devonian), Beecher’s Trilobite Bed (Ordovician), and the La Voulte-sur-Rhône marls (Jurassic).

## Introduction

**Fig 1:**
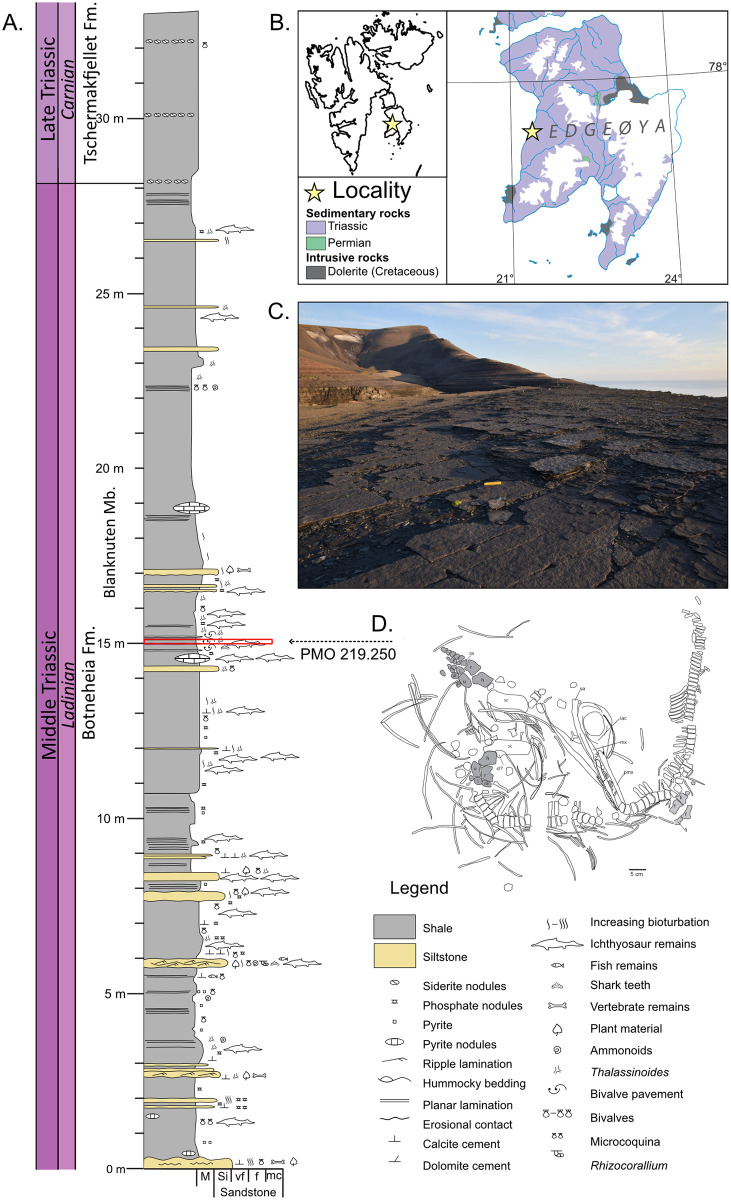
The study site at the Muen Mountain. **A**. Stratigraphical log of the Muen Mountain plateau. **B**. Geological map of the island Edgeøya, showing its placement in the Svalbard archipelago. The map of Edgeøya is reprinted from [9] under a CC BY license, with permission from Dallmann, original copyright 2015. **C**. The Muen plateau, bedding planes are exposed over large areas. In the front of the picture is the lower of three bivalve beds. **D**. Sketch of the specimen PMO 219.250 with its placement in the stratigraphy.

Paleontological sites with exceptionally preserved (Konservat) or particularly abundant (Konzentrat) fossils are known as ‘lagerstätten’ [1]. These sites are invaluable windows to the past, providing comprehensive views of biodiversity and ecosystems contrasting the usually scattered paleontological record. Some of the cIassical fossil lagerstätten are particularly well-suited to X-ray investigations. Of particular fame in this respect are the Devonian Hunsrück Shale in Germany [2–4]; the Ordovician Beecher’s Trilobite Bed in New York State [5,6], and the Jurassic La Voulte-sur-Rhône Lagerstätte in southwest France [7,8]. In these cases, X-ray contrast is caused by the pyritization of the fossils, with the high atomic weight of Fe in the iron sulphide giving strong X-ray attenuation. Here we report on unique fossil preservation from the Blanknuten Member of the Botneheia Formation, Middle Triassic (Ladinian) on the Muen mountain on Edgeøya, Svalbard (Fig 1). In these beds, numerous vertebrate fossils are pervasively permineralised by mainly baryte, which has high atomic weight and extremely high X-ray attenuation. It should be noted that this unique preservation is present in the majority of specimens from the upper Botneheia Formation from multiple localities.

In recent years, the study of fossils has been transformed by the application of X-ray technologies, especially X-ray computed tomography and synchrotron tomography [8,10–12]. Although simple radiography is commonly used on invertebrates (see Hohenstein [4] for a short review of the early history of X-rays in palaeontology), it is rarely used on vertebrates where computed tomography or synchrotron tomography is normally utilised. These imaging techniques require a density contrast between the fossil and the matrix, such as with pyritized fossils, calcite fossils in shales, and subfossil remains with air in internal cavities.

Remineralization of fossils can follow several modes of formation; most common among vertebrates is perhaps authigenic preservation (e.g., pseudomorphs), and duripartic (hard part) preservation (e.g., skeletal material) [13]. Different modes of substitution include permineralisation, where the original bone is replaced; pore infilling, where the bone trabeculae are infilled with a secondary mineral; and pseudomorphing–a partial to complete dissolution of a phosphatic phase and subsequent recrystallisation (such that an unrelated mineral takes the form of the fossil). The mineral component of bones, hydroxyapatite ((Ca_10_(PO_4_)_6_(OH)_2_), can be recrystallised early during diagenesis and some workers even suggest that most apatitic fossils are pseudomorphs [14]. Common replacing minerals that are observed here are fluorapatite (Ca_5_(PO_4_)_3_F), sphalerite (ZnS), baryte (BaSO_4_) and in some instances pyrite (FeS_2_), although this most often is seen as diagenetic mineral growth rather than bone replacement.

**Fig 2:**
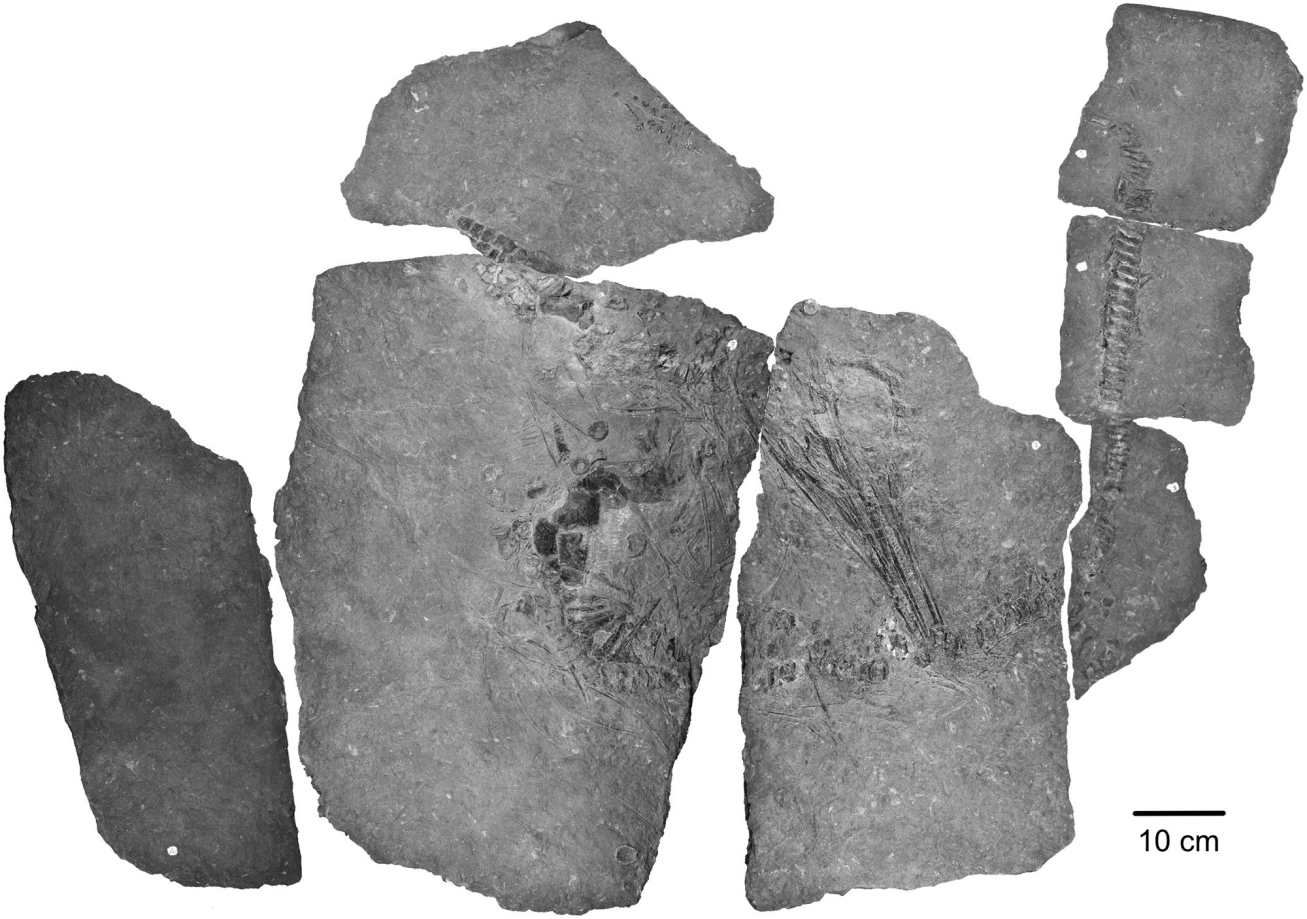
Photograph of PMO 219.250.

**Fig 3:**
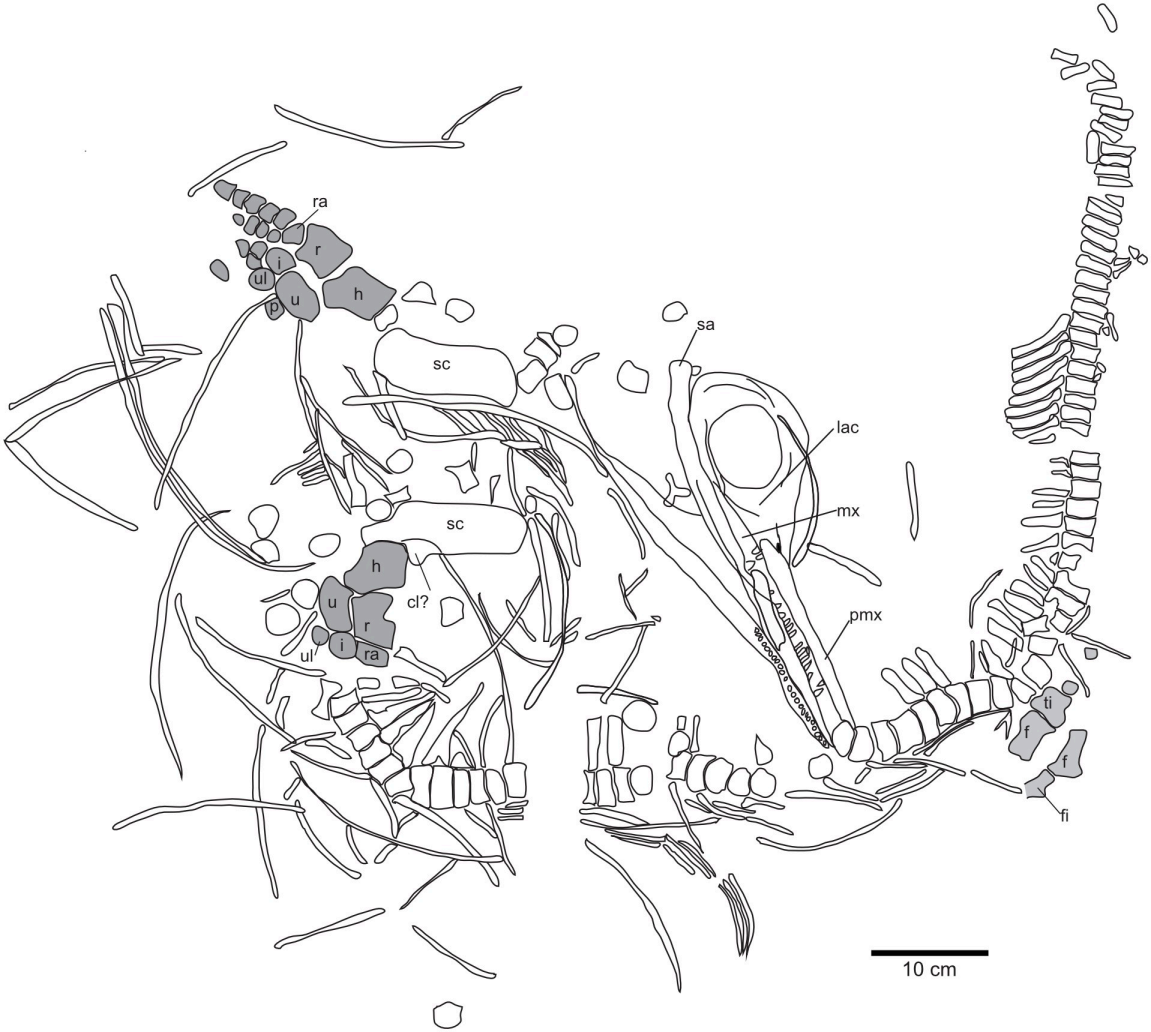
Schematic drawing of PMO 219.250. Abbreviations: cl-clavicula, f-femur, fi-fibula, h-humerus, i-intermedium, lac-lacrimal, mx-maxilla, pmx-premaxilla, r-radius, ra-radiale, sa-surangular, sc-scapula, ti-tibia, u-ulna, ul–ulnare. Modified from Hurum, Roberts [27].

The focus specimen of this study (PMO 219.250) (Figs 2 and 3) is a mixosaurid ichthyosaur from the Middle Triassic Botneheia Formation of Svalbard. Mixosauridae is a family of small-bodied ichthyosaurs found in Middle Triassic marine deposits from Svalbard, Italy, Germany, China, Switzerland and North America [15–18]. The two genera of mixosaurids are differentiated largely based off differences in dentition: *Phalarodon–*sports an ankylosed thecodont and usually heterodont dentition with bulbous crushing teeth posteriorly and conical teeth anteriorly; *Mixosaurus* has conical teeth set in grooves (subthecodont), tooth size may increase slightly posteriorly [19,20]. However, the number of valid taxa has been the subject of debate in the literature, with multiple taxa being reassigned to different genera [16,18,21,22]. Currently seven species are broadly accepted: *Mixosaurus cornalianus*, *Mixosaurus kuhnschyderi*, *Mixosaurus xindianensis*, *Phalarodon atavus*, *Phalarodon callawayi* and *Phalarodon fraasi* [22,23]. An additional species *Barracudasauroides panxianensis*, has either been seen as an outgroup to *Mixosaurus* and *Phalarodon* [24,25] or incorporated into the genus *Mixosaurus* [26]. Currently only *Phalarodon fraasi* and *Phalarodon callawayi* have been confirmed from the Middle Triassic of Svalbard [15,18,22,23,27].

**Fig 4:**
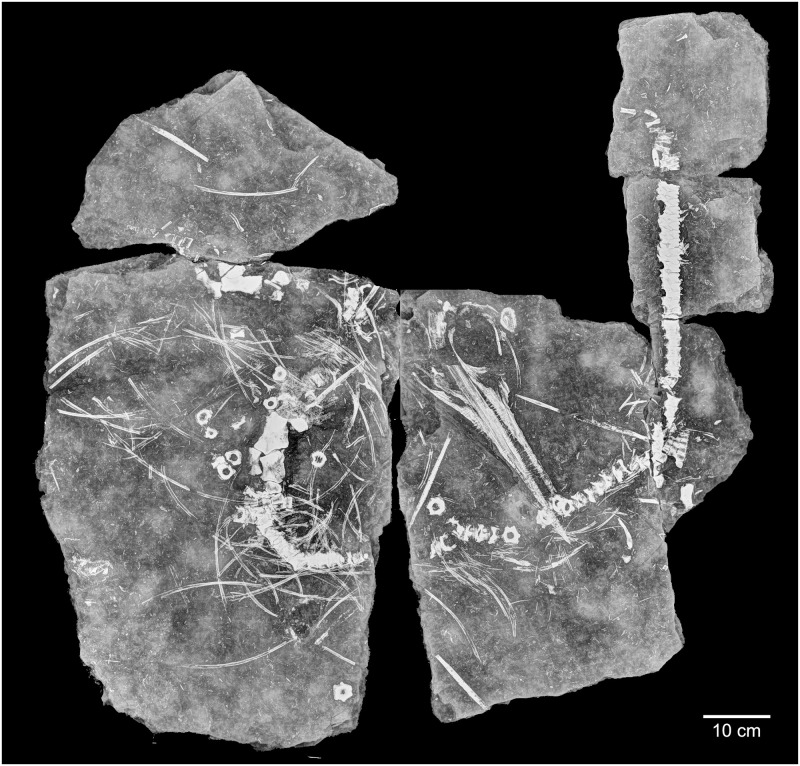
Radiograph image of PMO 219.250.

Initially, PMO 219.250 was tentatively referred to *Phalarodon*, based on having a pronounced narial shelf and maxillary dentition set in sockets [27]. During re-examination of the specimen in Økland, Delsett [22], this referral was questioned due to the placement of PMO 219.250 in *Mixosaurus* by phylogenetic analysis. However, this was weakly supported, forming a polytomy with the rest of *Mixosaurus*. Using the new radiographs of PMO 219.250 (Fig 4), details are now available to help resolve the taxonomic placement of this specimen.

## Geological setting

The Arctic Archipelago of Svalbard lies in the Arctic Ocean and consists of the main island Spitsbergen with several smaller islands. To the east lies Edgeøya (Fig 1A). Here, Triassic rocks crop out in most hillsides. In the Middle Triassic, Svalbard was a part of the Boreal Sea on the northern margin of Pangea. It opened into the Panthalassa Ocean and was a large open-marine shelf at approximately 45°N palaeolatitude. Deposition in the Middle Triassic was basinal, resulting in dark shales and occasional siltstone beds [28]. In the Middle Triassic, deposition in western Svalbard resulted in the shallow marine Bravaisberget Formation while the central and eastern parts record the deeper marine Botneheia Formation [29,30].

The Botneheia Formation is rich in organic material and well-studied as an onshore analogue to potential source rocks in the Barents Sea [e.g., 28,29,31–37]. The Botneheia Formation contains storm-originated siltstones and carbonate-cemented black shales [28]. Phosphate nodules are found throughout the interval, either dispersed in the shale or as basal lags in the siltstone [28,38]. The early Anisian saw a transgression that led to deep-shelf, anoxic depositional environments throughout the lower Muen Member, before conditions fluctuated more in the Ladinian part of the Blanknuten Member [29,30,36–38].

PMO 219.250 was collected from Edgeøya, Svalbard, as part of an excavation in 2008, but was not then placed in the stratigraphy [27]. Detailed stratigraphic field work was conducted later in 2018 on the Muen Mountain (77°49’24.2” N 21°22’39.4” E, 118 m a.s.l.) on Edgeøya, Svalbard (Fig 1). The Muen plateau is approximately 500 metres long and 40–70 metres wide. It is exposed stepwise with a total vertical thickness of only 120 cm. The studied section covers a characteristic part of the Blanknuten Member of the Botneheia Formation, containing three bivalve beds occuring within 30 cm of the stratigraphy (from 15 m to 15.3 m in the logged section, Fig 1A). These are almost monospecific to *Daonella degeeri*, and several cm thick. The beds are autochthonous and formed over periods of low sedimentation rates and winnowing. Ichthyosaur specimens are scattered and embedded into the beds with varying degrees of preservation from scattered single elements (PMO 234.170, PMO 234.171), to disarticulated specimens (PMO 234.168) as well as articulated specimens (PMO 219.250). Ichthyosaur specimens often weather in an iconic blue colour due to the iron phosphate mineral vivianite and are usually semi-articulated and flattened.

### Stratigraphic position of PMO 219.250

PMO 219.250 was initially correctly presented as collected from the Muen plateau on Edgeøya by Hurum, Roberts [27] (Fig 1B), but the placement of the locality in their Fig 1a was shown as Mudalen, 3 km NE of the Muen plateau. We place PMO 219.250 in the lowermost bivalve bed (Fig 1B). This is based on inspection by V.S.E. of the excavation site with one of the authors of the Hurum et al. 2014 study (A.M), and photographs from the excavation.

## Methods

### Institutional abbreviations

PMO Palaeontological Museum Oslo (presently: the Natural History Museum, University of Oslo).

### Ichthyosaur material

Material for this study was collected during fieldwork on Edgeøya, Svalbard (Fig 1A–1C) in 2008 (PMO 219.250) and 2018 (other ichthyosaur specimens, log and sampling). PMO 219.250 (Fig 1D) was described by Hurum, Roberts [27]. PMO 219.250 was initially mechanically prepared in an external laboratory [27], with experience on similar material. Although chemical preparation has not been attempted on PMO 219.250, methodology using 10% acetic acid was tested on mixosaurid material from the same formation in Økland, Delsett [22]. This had limited success on specimens embedded in shale matrix.

### Specimen selection

When selecting specimens for this study, samples were first radiographed to identify specimens with good contrast. These were then CT scanned to obtain three-dimensional models before they were cut to make thin sections. In specimens with a density contrast, using X-ray imaging before any destructive analysis can help identify areas of interest and possibly reduce the destructive analysis methods needed. X-rays do not damage the specimens, and it is relatively quick to determine if there is sufficient contrast.

### Radiograph

The radiograph of PMO 219.250 was made with a General Electric X-ray apparatus at the Museum of Cultural History, University of Oslo, at 110 kV, 2 mA, and 10 s exposure time. The phosphor cassette was scanned with a GE CRxFlex scanner at 50 μm pixel resolution. Adobe Photoshop was used to align and merge images. Adobe Illustrator was used to place individual slabs in their original position based of their original description [27].

### Computer tomography

Specimens used for thin sections were CT scanned before they were cut. Computer Tomography (CT) scans and radiographs of smaller specimens were conducted using a Nikon Metrology XT H4 225 ST microfocus CT at the Natural History Museum in Oslo, with varying scanning parameters. Images were processed with Avizo 2020.2. Samples were CT scanned to identify areas of interest before these were cut into thin sections. The high content of pyrite and/or baryte in the fossil remains gave excellent contrast, making it possible to identify ammonoids and bivalves within the sediment.

### EDX and SEM point measurements

Semi-quantitative elemental analysis was performed on thin sections. Analyses were conducted using a Variable Pressure Hitachi S–3600N Scanning Electron Microscope (SEM) equipped with a backscattered (BSE) electron detector and a Bruker XFlash^®^ 5030 energy dispersive X-ray detector (EDX). The software used was Esprit 1.9. All analyses were conducted in low vacuum mode (15 Pa) and the acceleration voltage was 15 kV.

### Thin sections

To avoid destructive sampling of PMO 219.250, thin sections were prepared from ichthyosaur specimens PMO 234.168 and PMO 234.170, collected from the same bivalve bed as PMO 219.250. PMO 234.171 from the bivalve bed above, and PMO 234.169 from the microcoquina bed below, were also included in the study. Thin sections were prepared by the thin section workshop at the Department of Geosciences, University of Oslo. The pre-cut cubes were impregnated using the epoxy EpoFix Resin. CaldoFix was used to attach the sample to a glass plate, and the sample was then cut with a diamond microsaw. Coarse polishing was done using a Logitech polishing machine and Buehler phoenix 4000 polishing machine. A Thorlag grinding and polishing automat with increasingly finer polishing paper was used for the fine polishing. Images of thin sections were taken with a Leica DMPL microscope with a Leica MC 170 HD camera.

## Results

The excellent X-ray contrast in the Botneheia Formation is mainly caused by baryte permineralisation and pseudomorphing. Relatively few minerals were found in this study, mainly carbonate (CaCO_3_), dolomite (CaMg(CO_3_)_2_), fluorapatite (Ca_5_(PO_4_)_3_F), pyrite (FeS_2_), baryte (BaSO_4_), and sphalerite (ZnS). Sphalerite occurs both amorphous (PMO 234.168) or as crystals within baryte (PMO 234.170, PMO 234.171), while the baryte occurs as either completely recrystallised pseudomorphs or as permineralisation of trabecular bone. Pyrite in the matrix around the ichthyosaur specimens is mainly seen as either cubic or framboidal grains. Framboidal pyrite growth (and the combination with or lack of cubic grains) is indicative for the redox conditions at deposition [39]. Pyrite also sometimes forms a rim on the outside of the remineralised bone, similar to that reported by Wings [40] from multiple English Mesozoic vertebrate assemblages. No celestite (strontium sulphate, SrSO_4_) was found, although trace peaks for Sr occurred in some of the EDS point measurements. Celestite is however mainly known from evaporitic facies [41,42].

### Radiograph

**Fig 5:**
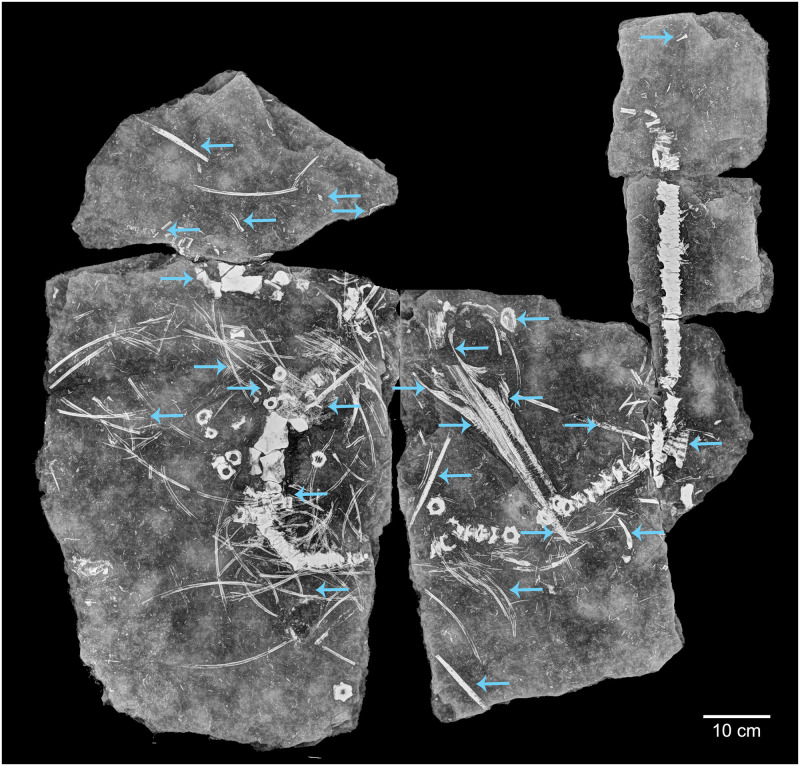
Radiograph of PMO 219.250. Blue arrows indicate details not seen on the actual specimen, but visible on the radiograph.

**Fig 6:**
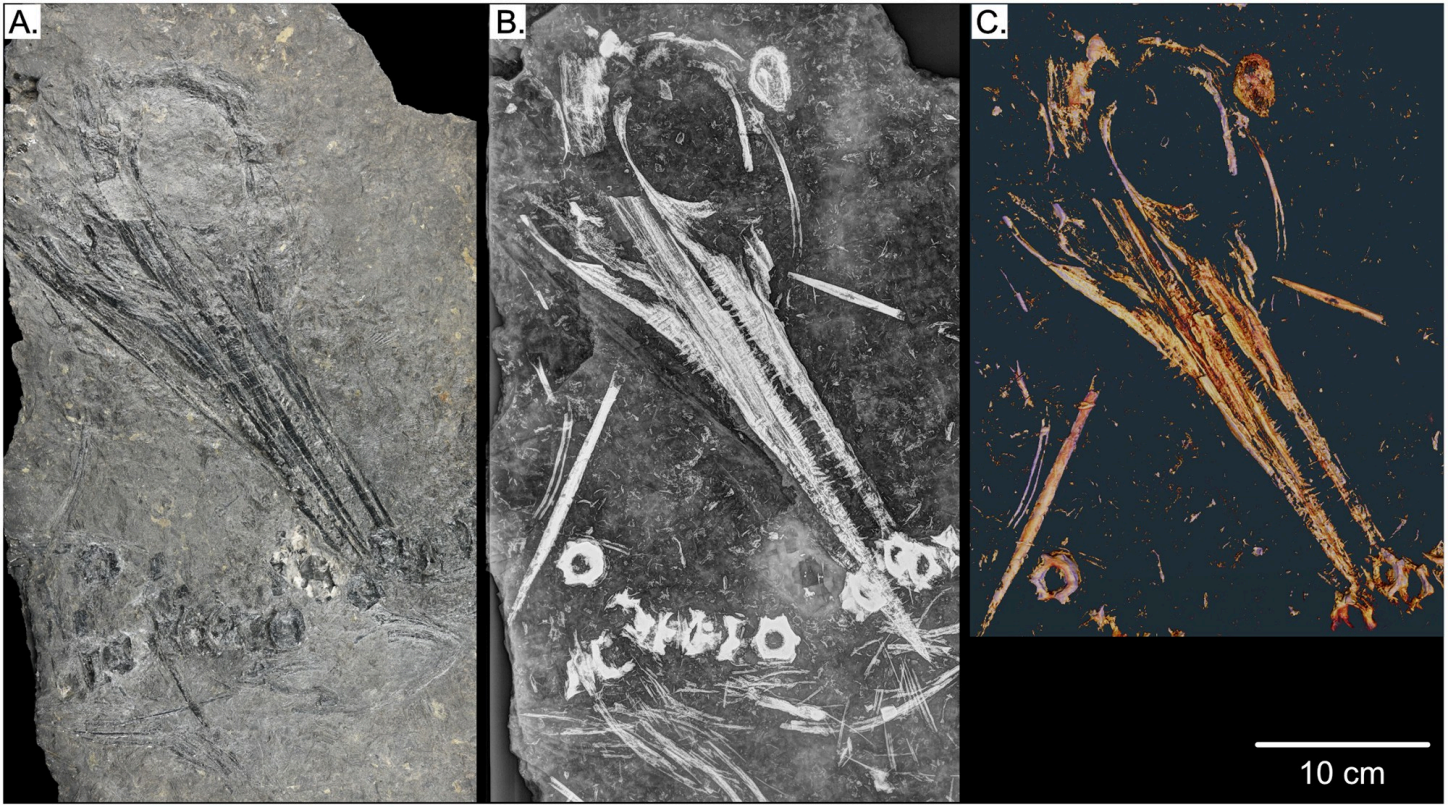
Method comparison on the skull of PMO 219.250. A. Photograph (visual observation). B. Radiograph. C. Computed Tomography scan.

Due to the strong density difference between bone and matrix, the radiograph of PMO 219.250 provides excellent detail revealing fine details otherwise hidden within the sediment (Figs 2–6; see S1 Fig for a more typical contrast difference). In addition to several unidentified elements, both the right forefin (Fig 3, upper left) and right hind fin (Fig 3, femur closest to the vertebral column) revealed more articulated carpal/tarsal elements. The right forefin appears to have the distalmost partially articulated carpals preserved, giving a length estimate of approximately 13 cm from the ulnar facet, and 22 cm measured from the proximal end of the humerus. Teeth in the left premaxilla can be seen underneath the right dentary, revealing more teeth than previously described [27]. The posterior dentary teeth are more robust than the anterior ones, as already described by Hurum, Roberts [27], although not to the degree as in other contemporaneous Svalbard specimens [22,23]. The roots of the maxillary and dentary teeth are clearly visible and display a clear groove in larger teeth (see Discussion). The angular/surangular is clearly visible. The dentary snout tip is preserved and visible in the radiograph, for the first time providing a total skull length of approximately 41 cm, significantly longer than the original estimate of 28 cm [27]. Certain skull sutures are surprisingly visible in the radiograph, such as the contacts between the jugal and lacrimal, and the nasal and premaxilla. This allows further interpretation of the borders of the external naris, which appears to be dorsally and ventrally formed by the nasal and maxilla respectively. The premaxilla appears excluded from the external naris, differing from the original description where it could not be described [27]. It is also clear that the posterior extent of the maxilla does not surpass the anterior rim of the orbit, unlike some mixosaurid species [18]. PMO 219.250 has strings of vertebrae preserved, and it is possible to see the intervertebral spacing between them. Interestingly, not all bones were visible in the radiograph. Some girdle elements, such as the two scapulae and the right femur and tibia, are not seen at all in the radiograph as most of the bone is eroded with little material remaining. Associated fossils that were only discovered on the radiograph are the fragmented remains of a teleost fish (vertebrae and ribs) and two belemnoids (approximately 15 and 21 cm long). Such Middle Triassic belemnoids are usually referred to the waste basket taxon *Atractites* [43].

### Sulphide and sulphate infilling

**Fig 7:**
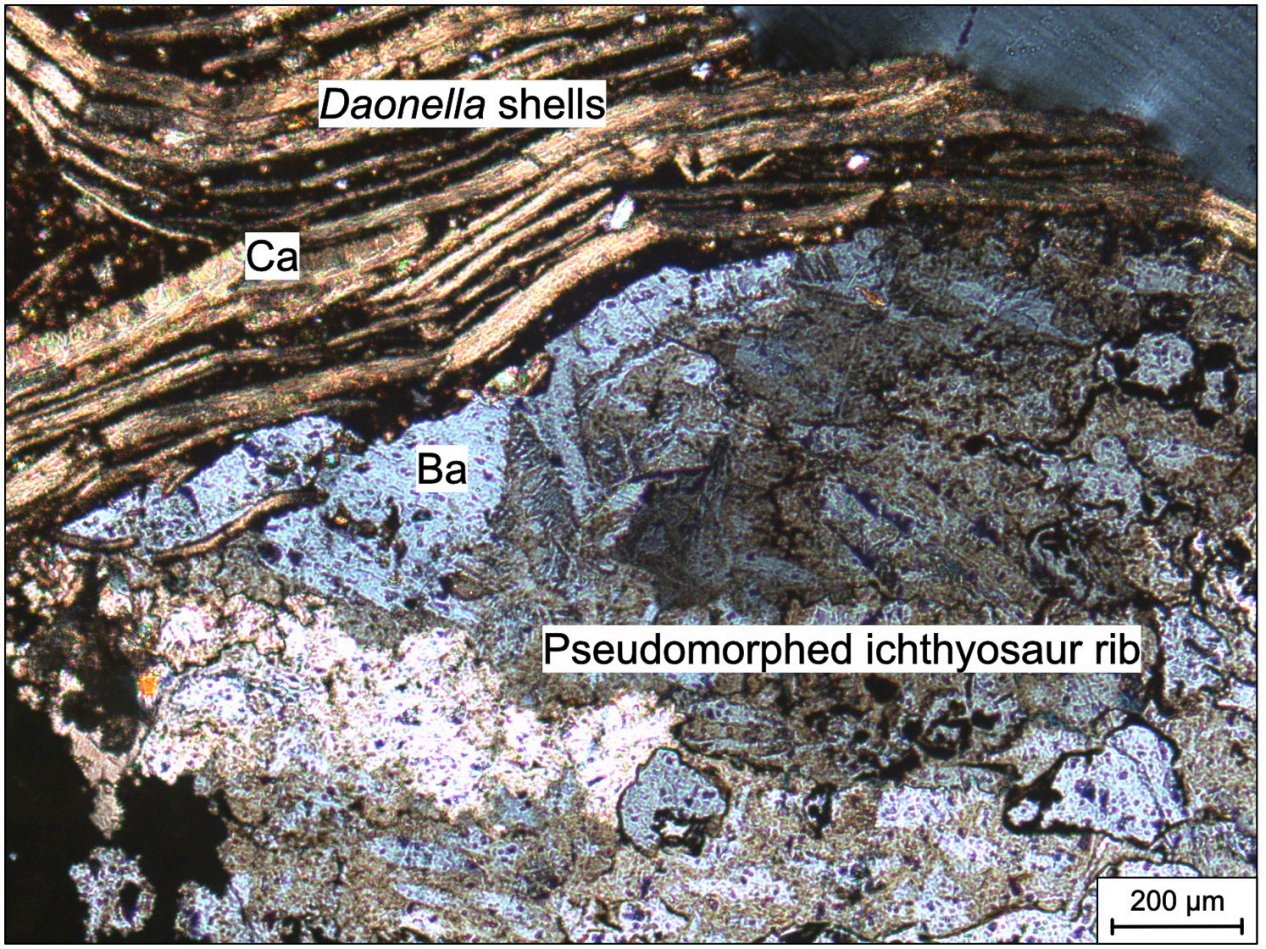
Photomicrograph of PMO 234.168 in polarised light. Bivalve shells surround the rib that is completely replaced by baryte.

Sulphide and sulphate minerals were identified by Electron Diffraction Scanning (EDS) maps of thin sections (S2 Fig), and then by using point measurements (S3 Fig) to infer mineralogy more precisely. EDS is not sensitive to small amounts (~2–5%) of ions in the crystal lattice [40]. We could therefore not determine potential trace elements in the sulphides and sulphates. Optical microscope analysis revealed the characteristic blue interference colour of baryte (Fig 7). However, as bone material is also replaced by sulphides and as most of these (such as pyrite and sphalerite) are opaque, Scanning Electron Microscope techniques proved more useful for the study.

### Thin sections

**Fig 8:**
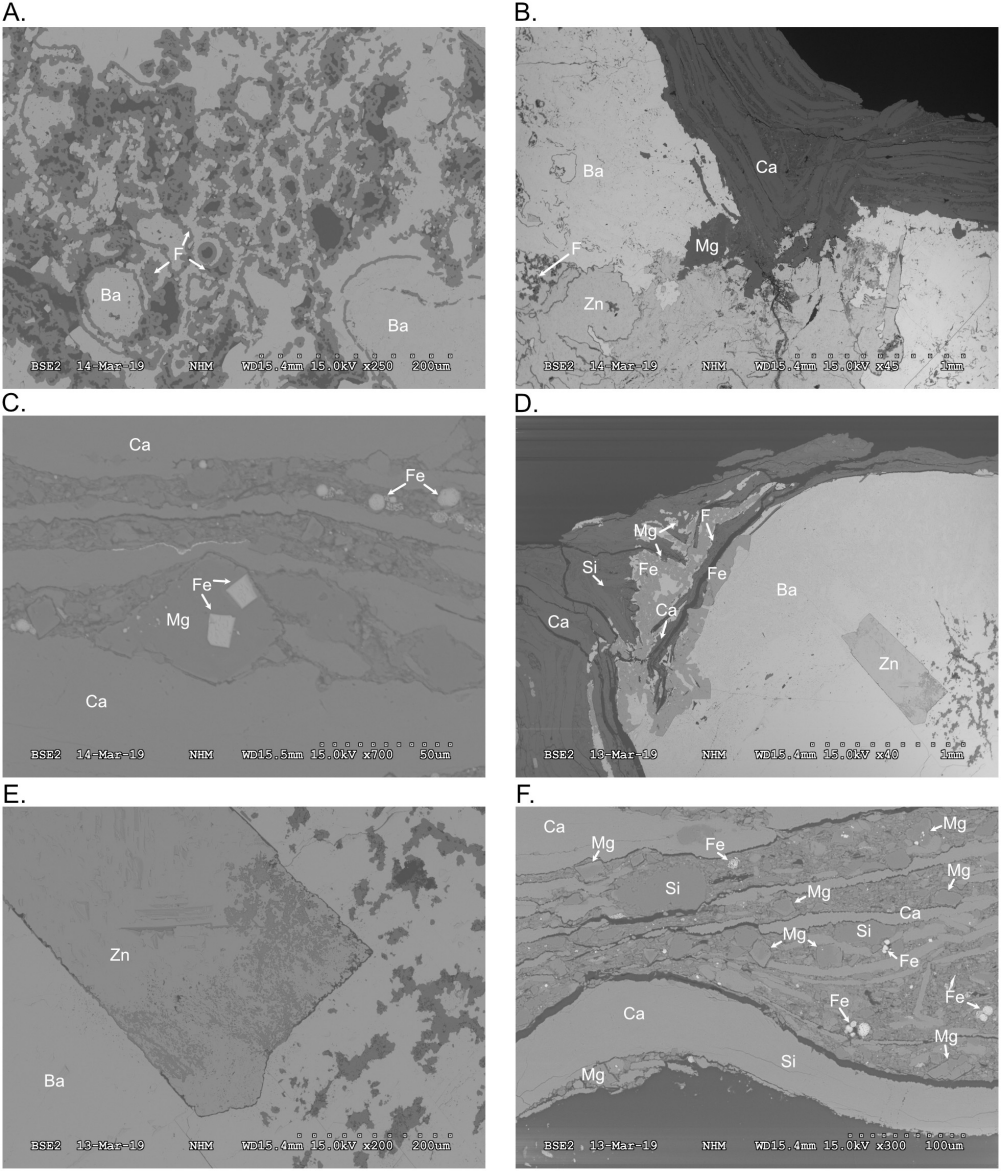
Scanning Electron Microscope (SEM) images of PMO 234.168 and 234.170. **A**. Remnants of bone trabeculae in rib from PMO 234.168. **B**. Contact point between rib and matrix. **C**. PMO 234.168, cubic and framboidal pyrite within the lower bivalve bed. **D**. PMO 234.170, rib replaced by baryte and sphalerite crystal. The surrounding matrix is carbonate with dolomite and quartz grains dispersed. **E**. PMO 234.170, close-up of contact between sphalerite crystal and baryte. **F**. PMO 234.170, large bivalve shell in the bottom of the image, with layers of thinner bivalve shells and angular dolomite grains above. Framboidal pyrite is scattered throughout the matrix. Abbreviations: Ba = baryte, Ca = carbonate, Mg = dolomite, F = fluorapatite, Fe = pyrite, Si = quartz, Zn = sphalerite.

PMO 234.168 (Fig 8A and 8B) consists of a rib embedded in planar layers of the lower bivalve bed. Mainly calcitic amorphous matrix is dispersed in between the bedding. Certain layers resemble calcite seams more than layers of bivalve shells. Pyrite is present both in cubic and framboidal form (Fig 8C). The rib contains both remnants of original bone structure permineralised by baryte and with fluorapatite in the pore wall, and amorphous pseudomorphing by both baryte and sphalerite.

PMO 234.170 is an ichthyosaur rib on top of distorted layers of the lower bivalve bed (Fig 8D). The matrix surrounding the rib is dolomite and carbonate, with pyrite and baryte at the contact. There are also remains of flourapatite in the contact area, but most of the rib is completely replaced by baryte. A large (~720 μm long, 280 μm wide) sphalerite crystal is seen in the middle of the remineralised rib (Fig 8E). Carbonate bivalve shells lie in a matrix of dolomite (Fig 8F).

**Fig 9:**
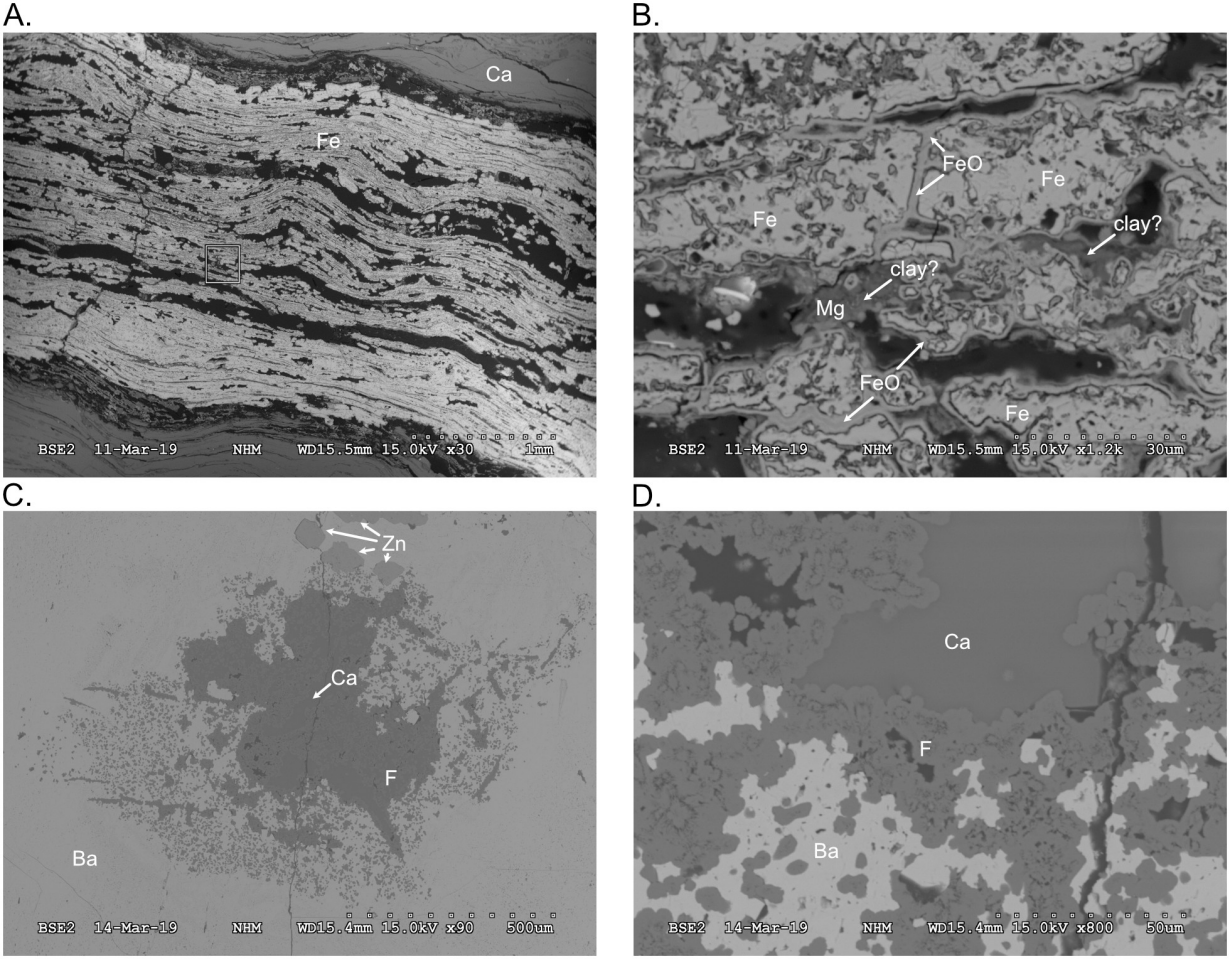
Scanning Electron Microscope (SEM) images of PMO 234.169 and 234.171. **A**. PMO 234.169, pyritized ammonoid in microcoquina. **B**. Close-up of ammonoid. The pyrite has a rim of iron oxide. Cavities are filled with dolomite grains and unidentified clay minerals. **C**. PMO 234.171, centre of rib with a centre of carbonate, surrounded by amorphous fluorapatite and embedded in baryte. Sphalerite crystals are seen in the top of the image. **D**. PMO 234.171, close-up of centre of rib. Fluorapatite network filled with carbonate and surrounded by baryte. Abbreviations: Ba = baryte, Ca = carbonate, F = fluorapatite, Fe = pyrite, FeO = iron oxide, Mg = dolomite, Zn = sphalerite.

PMO 234.169 is a microcoquina. Layers are distorted: while some are flat, most are deformed as z-folds. PMO 234.169 contains a pyritised ammonoid (Fig 9A), with an oxidation rim around the edges (Fig 9B). Pyrite is both cubic and framboidal. No other sulphur minerals were present in the sample. Interestingly, the ammonoid was only discovered during the CT-scanning of the specimen, as it was within the sample.

PMO 234.171 is a rib embedded in the bivalve bed above the lowermost bivalve bed. The main replacing mineral is baryte, with a rim of pyrite around the rib. The matrix is carbonate and dolomite. In the central part of the rib, remnants of the bone structure is visible (Fig 9C and 9D). The pores are filled with carbonate and surrounded by baryte (Fig 9D). Amorphous baryte surrounds the flourapatite.

### Bivalve beds as sulphur ducts

Both PMO 234.168 from the lower bivalve beds and PMO 234.171 from the bivalve bed above are almost completely replaced by baryte. This is not seen in PMO 234.169 from the microcoquina bed. It has been suggested that the bivalve beds have acted as ducts for sulphidic fluids, as has been described for the Lower Oxford Clay [44]. Comparison of the bivalve beds and the microcoquina is hindered by the lack of bone material in PMO 234.169 from the microcoquina. However, the bivalve beds also show extensive pyritization as would be expected in sulphur ducts, which is not seen in the microcoquina.

## Discussion

PMO 234.168 is a semi-articulated mixosaurid specimen, and PMO 234.170 is an ichthyosaurian rib. Both PMO 234.170 and PMO 234.168 showed: 1) original bone structure with pores and trabeculae consisting of fluorapatite and/or permineralised by baryte, 2) areas of total replacement by amorphous baryte with crystalline sphalerite, and 3) rims of pyrite. CT scanning of PMO 234.168 gave the same excellent contrast as for PMO 219.250. We consider both specimens to be good equivalents for discussing the preservation of PMO 219.250.

### Baryte provides extreme X-ray contrast

The baryte preservation of vertebrate fossils in the Botneheia Formation gives extremely high X-ray contrast and this is a main reason for the X-ray imaging quality. In fact, baryte has a linear X-ray attenuation coefficient of 48.1 cm^-1^, compared with only 7.1 cm^-1^ for even pyrite [45], which is responsible for the X-ray contrast in e.g., the Hunsrück shale.

### Baryte in both the Triassic and the Jurassic of Svalbard

Baryte can precipitate in the water column in association with the decay of organic matter [46,47], and baryte in sediments has therefore been used as a paleoproductivity indicator [e.g., 48]. Baryte may also form in connection with hydrothermal activity and by other abiotic processes [49]. Pervasive baryte permineralisation in bone has previously been reported in the Upper Jurassic Slottsmøya Member of the Agardhfjellet Formation in Central Spitsbergen, Svalbard [50–52]. High levels of barium in the surrounding shale [52] and the presence of paleoseeps with authigenic carbonate [53] prompted Delsett, Novis [51] to suggest that seep-related methanogenesis could have remobilized barium by reduction of porewater sulphate, leading to undersaturation of sulphur and dissolution of baryte [cf. 54].

### Mineralisation processes

Baryte permineralisation of bone is unusual, especially in non-evaporitic facies. Our new finds of complete pseudomorphing of bone by baryte in the Middle Triassic of Svalbard differs from the Late Jurassic permineralisation. Complete sphalerite crystals within ribs that are almost completely replaced by baryte, suggests that vertebrate bones could have functioned as fluid ducts where crystals could precipitate. Sphalerite crystallises between 375 degrees and 460 degrees °C [55], while baryte crystallises down to temperatures of between 120 and 240°C in hydrothermal vents [56] and between 10 and 70°C at surface pressure [57]. The high crystallisation temperature of sphalerite is close to the T_max_ calculated for the Botneheia Formation in Edgeøya; between 440 and 447°C at Blanknuten, approximately 20 km north of the Muen mountain [58]. The three mineralisation processes are likely to have happened consecutively, first as hydroxyapatite in bone trabeculae was replaced by baryte, possibly due to dissolution and mobilisation of barium from the surrounding shales as described for the Late Jurassic by Delsett, Novis [51]. Later, as the bone was dissolved, sphalerite crystals precipitated before the sulphur-rich fluids changed from reducing to oxidizing and amorphous baryte crystallised in the resulting cavity. Pyrite is often found as rims surrounding the baryte pseudomorphs, and this pyritization is thought to have happened latest.

### Differences in the baryte replacement in Jurassic and Triassic strata

Sulphidic fluids are linked to volcanism, and a regional, post-Jurassic event could have been responsible for the significant baryte mobilisation in organic-rich shales and precipitation into bone material on Svalbard. The Jurassic specimens on Central Spitsbergen were found more than one thousand metres higher up in the stratigraphy and at approximately 140 km distance from the locality described here. A volcanic event of such a large-scale is the High Arctic Large Igneous Province (HALIP). A Cretaceous (80–130 Ma) igneous province with sill emplacement into mainly Permian and Triassic rocks caused heating and hydrothermal alteration of the Triassic to earliest Cretaceous sediments in Svalbard [59]. However, an unpublished study found that the sills were preferentially emplaced in the lower mechanical competence lithologies of the Botneheia Formation and Janusfjellet Subgroup [60]. We consider this a candidate event for the barium mobilisation in the Middle Triassic and suggest that it could be a contributing factor to barium migration in the Late Jurassic, in addition to cold methane seepage [51].

Compaction of Late Jurassic bone in Svalbard seems to have been partly hindered by the baryte permineralization [51]. Middle Triassic fossils are generally more flattened, especially in the bivalve beds. It is considered probable that this is due to the Triassic interval being buried significantly deeper than the Upper Jurassic at the time of baryte precipitation. However, a significant proportion of Early and Middle Triassic fossils are three-dimensional, which can be explained by lithological differences such as variations between shale, siltstone, and sandstone. Baryte has therefore likely been a stronger control for compaction in the lithologically more homogenous Upper Jurassic shale strata than in the more heterogenous Triassic strata. Also, bone material in the Upper Jurassic is permineralised, while specimens in this study have a combination of permineralised bone and pseudomorphs. The complete dissolution and recrystallisation of bone material in the Triassic can perhaps be linked to the deeper burial and subsequently higher temperatures. This is supported by the lack of sphalerite in the Jurassic specimens, as these did perhaps not undergo alteration at high enough temperatures for Zn to be dissolved in fluid and subsequently crystallise.

### New taxonomic information for PMO 219.250 from the radiographs

**Fig 10:**
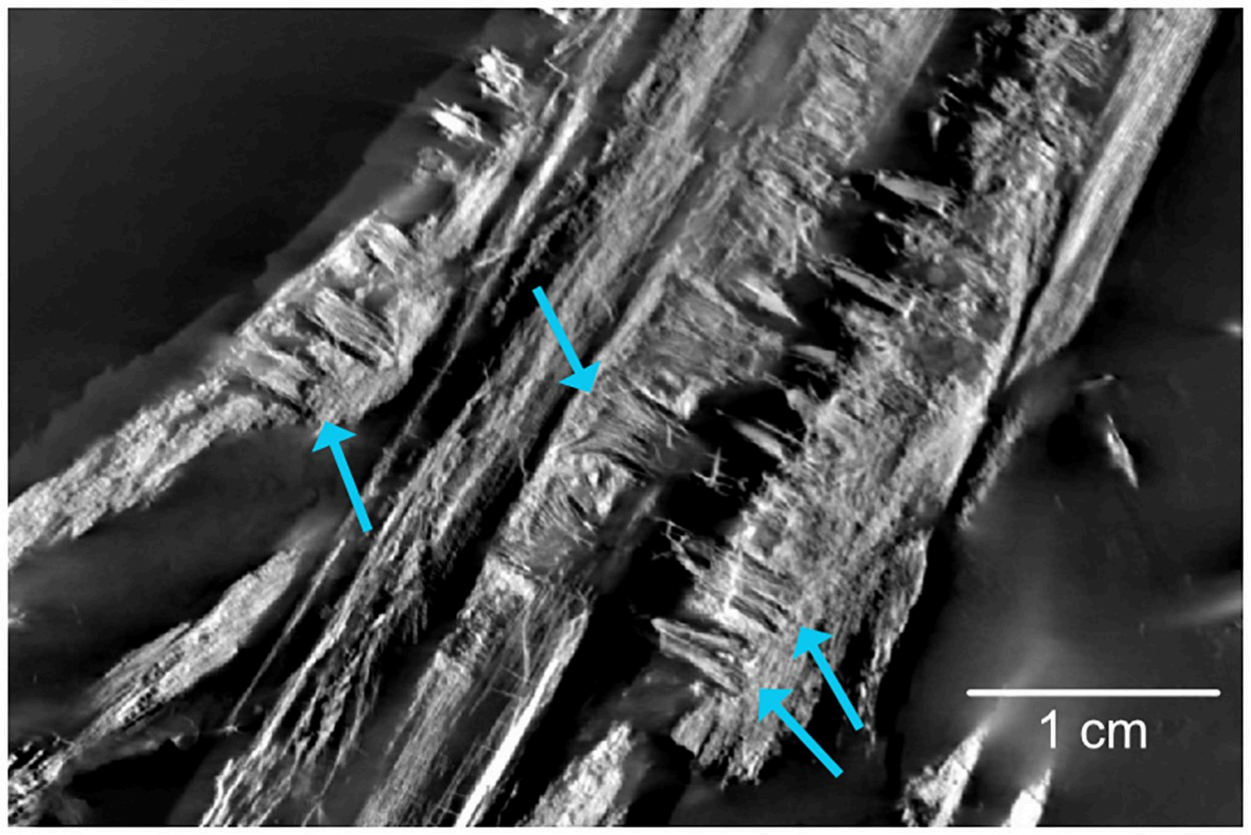
Detailed radiograph of the dentition of PMO 219.250. Blue arrows indicate where grooves in the tooth roots can be observed.

Utilising computed tomography is a common method of increasing the amount of taxonomic information that can be extracted from fossil specimens [e.g., 61,62]. This allows for areas to be examined in detail that cannot be prepared due to specimen preservation, as well as providing details hidden by other bone elements [63]. The fragility and compressed nature of PMO 219.250 as well as the composition of the matrix, does not allow for further preparation of this specimen mechanically or chemically without damage. The new radiographs of the specimen display many new features, previously not observed. Among these, the most essential for taxonomic identification are the detailed images of the dentition of the specimen (Fig 10). There is a minor differentiation between anterior and posterior teeth: anteriorly, the teeth set in the dentary, premaxilla and maxilla are conical, tall and end in a point; whereas posteriorly (maxilla and dentary only) they are larger and bear a more blunted apex, particularly in the dentary. The dentition of the maxilla and dentary is set in sockets (anklyosed thecodont, sensu Motani [19]), corresponding with that seen in *Phalarodon* [64]. This differs from *Mixosaurus*, where the dentition is set in grooves (subthecodont; Motani [20]). As in *Phalarodon atavus* [16], no large posterior crushing teeth in the maxilla or dentary were observed in PMO 219.250, as seen in *Phalarodon fraasi* [22,23] and *Phalarodon callawayi* [18]. Similar to *P*. *atavus* (LPV 30872 –Liu, Motani [16], there is no constriction between root and crown, as observed in *Phalarodon cf*. *P*. *fraasi* [64]. The roots form around half of total tooth height as in *P*. *fraasi* [64]. However, the roots in the posterior portion of the dentary were significantly larger and more widely set (oval, not significantly labiolingually compressed) than anteriorly. In addition, a deep groove is present on the roots of larger teeth (see Fig 10) corresponding to that observed in *P*. *atavus*, an important synapomorphy for this species [17]. These important dental features suggest that PMO 219.250 is referrable to *Phalarodon* as suggested in the original description [27] and likely belongs to *P*. *avatus* based on the lack of clear heterodonty and the presence of a root groove on larger teeth.

## Conclusions

Specimens from the Arctic are difficult and expensive to collect, and many were collected in the early 20^th^ century. We wish to highlight X-ray techniques for studying specimens found from high X-ray contrast localities and to promote its use on museum specimens. Radiography and computed tomography can reveal details otherwise not sees in specimens, and has been increasingly used over the past decades [see examples in 12]. In specimen PMO 219.250, skull sutures and dentition were much clearer on the radiograph and CT than what could be observed by the naked eye, providing new information on the position of the external naris and dentition. Our new observations support the referral of PMO 219.250 to *Phalarodon* by Hurum, Roberts [27] and further suggest an affinity to *P*. *atavus*. As most other mixosaurids, specimens from the Botneheia Formation are flattened, semi-articulated and rarely preserved uncompressed [23], which causes significant loss of taxonomic information and often causes problematic phylogenetic relationships [22,23]. As demonstrated here, using radiography helps resolve the taxonomic identification of compressed mixosaurids from the Botneheia Formation. Although the Botneheia Formation specimens provides extreme X-ray contrast due to the unusual preservation, we hope that this methodology will be utilised further and may be applicable on compressed specimens from other localities.

## Supporting information

S1 Fig CT scan of the three-dimensional skull of a *Phalarodon fraasi* (PMO 235.393). Described by Roberts, Engelschiøn [23], showing the more typical contrast between fossil and matrix in the Botneheia Formation.
(TIF)

S2 Fig Examples of Energy Dispersive Spectrometer (EDS) elemental mapping. A. Remnant bone structure of rib in PMO 234.168. B. Contact between carbonate matrix (dark) and rib replaced by baryte and sphalerite. C. Pyrite precipitation in contact zone between baryte replaced rib in PMO 234.170 and carbonaceous matrix. D. PMO 234.170, carbonate bivalve shells (grey), with quartz grains and dolomitic rhombs.
(TIF)

S3 Fig Examples of Energy Dispersive Spectrometer (EDS) point measurements from PMO 234.168 showing different mineral spectra. A. Sphalerite. B. Fluorapatite. C. Baryte. D. Pyrite.
(TIF)

S1 Table Energy Dispersive X-ray Spectroscopy (EDS) point measurements of PMO 234.168. (XLSX)

S2 Table Energy Dispersive X-ray Spectroscopy (EDS) point measurements of PMO 234.170. (XLSX)

S3 Table Energy Dispersive X-ray Spectroscopy (EDS) point measurements of PMO 234.169. (XLSX)

S4 Table Energy Dispersive X-ray Spectroscopy (EDS) point measurements of PMO 234.171. (XLSX)
