# Supplementary figures and images for: Exceptional X-Ray contrast: Radiography imaging of a Middle Triassic mixosaurid from Svalbard
Source: PLoS One. 2023 May 31;18(5):e0285939. doi: 10.1371/journal.pone.0285939 (PMC10231774; doi:10.1371/journal.pone.0285939)

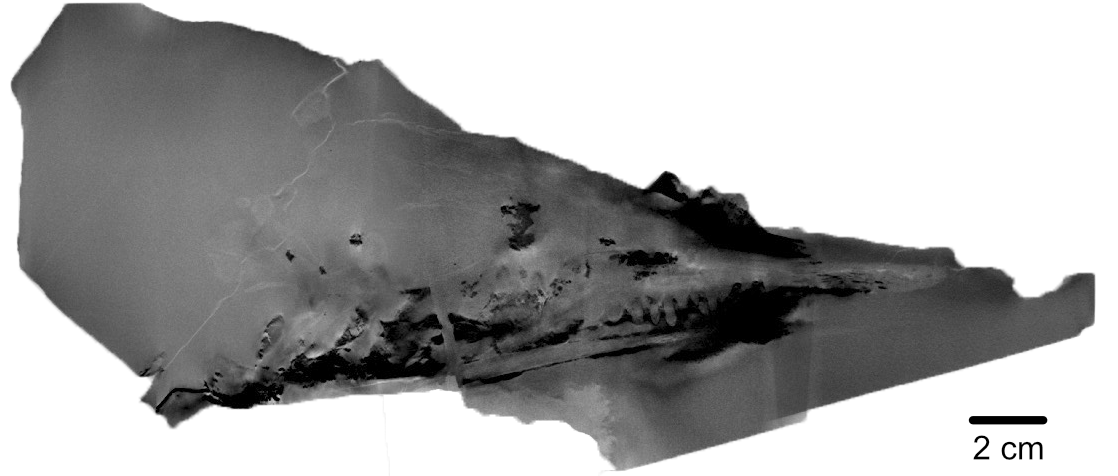

Supplement: S1 Fig — Described by Roberts, Engelschiøn [23], showing the more typical contrast between fossil and matrix in the Botneheia Formation. (TIF) [file pone.0285939.s001.tif]

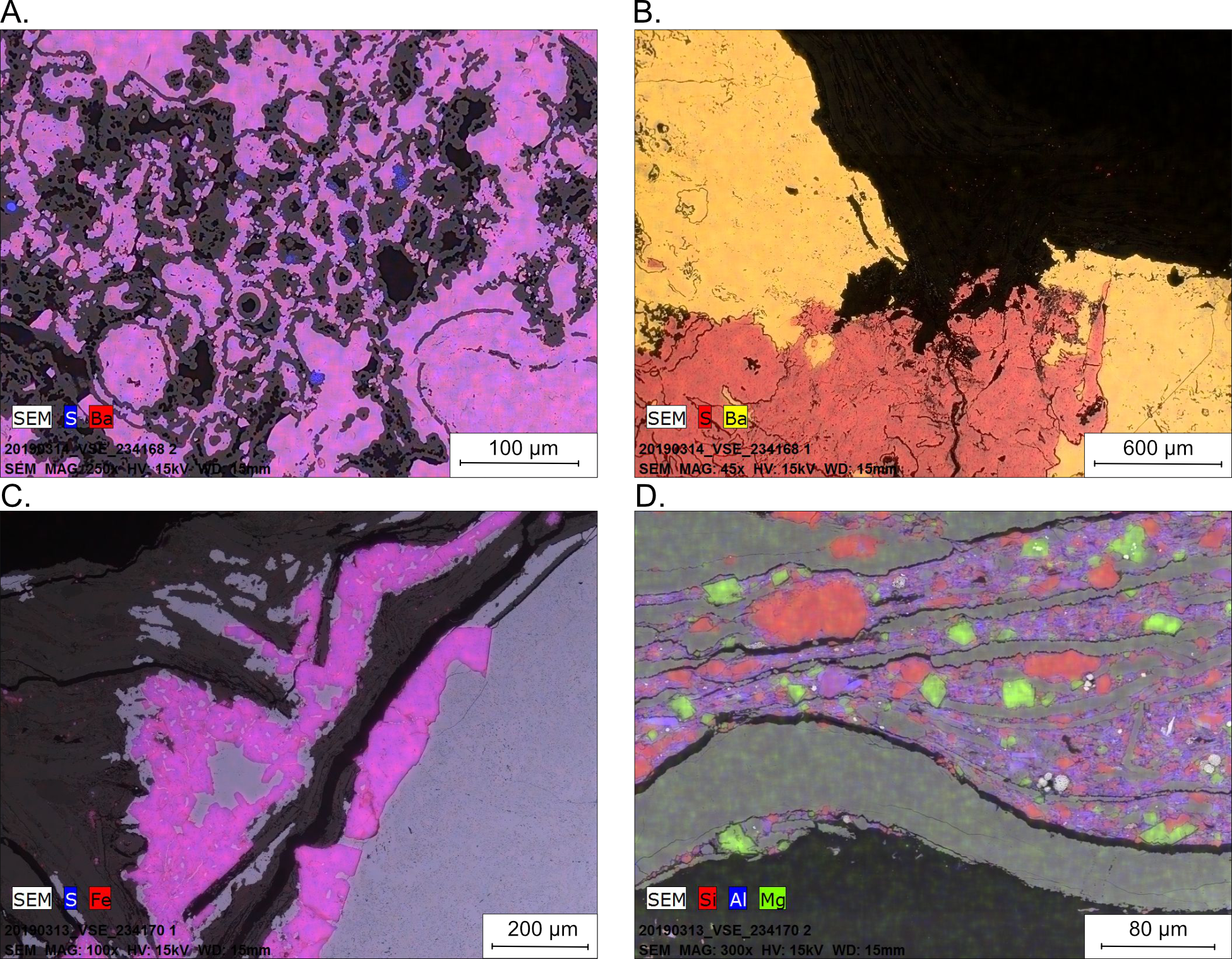

Supplement: S2 Fig — A. Remnant bone structure of rib in PMO 234.168. B. Contact between carbonate matrix (dark) and rib replaced by baryte and sphalerite. C. Pyrite precipitation in contact zone between baryte replaced rib in PMO 234.170 and carbonaceous matrix. D. PMO 234.170, carbonate bivalve shells (grey), with quartz grains and dolomitic rhombs. (TIF) [file pone.0285939.s002.tif]

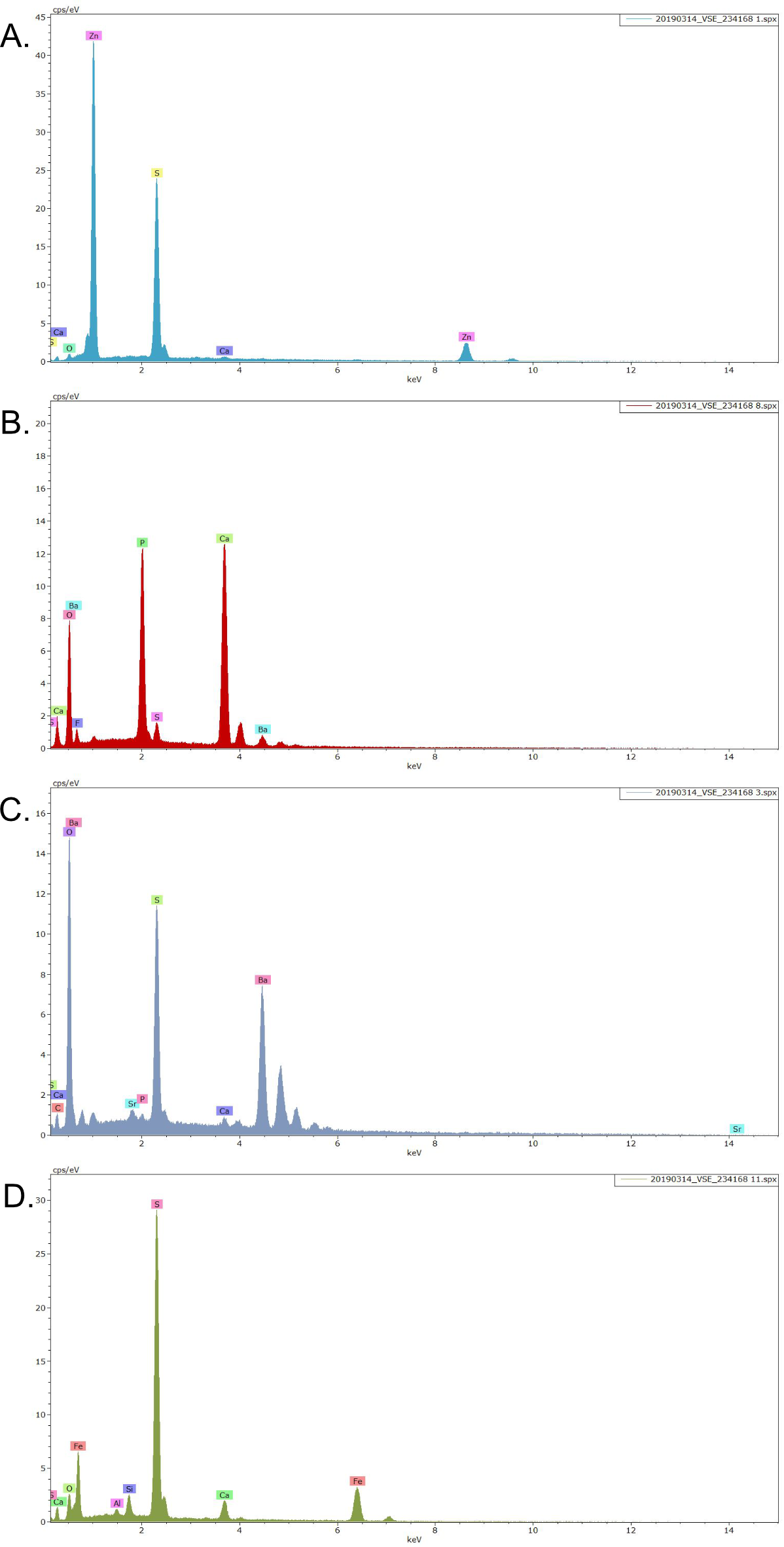

Supplement: S3 Fig — A. Sphalerite. B. Fluorapatite. C. Baryte. D. Pyrite. (TIF) [file pone.0285939.s003.tif]
